# Lawn or spontaneous groundcover? Residents’ perceptions of and preferences for alternative lawns in Xianyang, China

**DOI:** 10.3389/fpsyg.2023.1259920

**Published:** 2023-10-27

**Authors:** Huiyi Liang, Cangshuan Li, Denggao Xue, Jiangnan Liu, Kedi Jin, Yuebin Wang, Dongyang Gao, Yingyuan Chen, Yapeng Li, Ling Qiu, Tian Gao

**Affiliations:** ^1^College of Landscape Architecture and Art, Northwest A&F University, Xianyang, China; ^2^Forestry Science Institute of Xianyang, Xianyang, China

**Keywords:** landscape perception, landscape preference, spontaneous vegetation, urban lawn, social background

## Abstract

Within urban green spaces, spontaneous groundcovers, as potential alternatives for traditional lawns, have garnered attention due to their ecological adaptability. However, little attention has been paid to whether spontaneous groundcovers can serve as suitable replacements for lawns in terms of the aesthetic values and human preferences for each. Based on questionnaires accompanied by photo elicitation, this study explored the perceptions of and preferences for seven kinds of lawns and six kinds of spontaneous groundcovers in China. The effects of social backgrounds on people’s perceptions of and preferences for ground covers were also analyzed. The results indicated a general equivalence in preferences for the lawn and spontaneous groundcover. The *Taraxacum mongolicum – Cynodon dactylon – Conyza canadensis* community was significantly preferred most among all of the selected ground covers. Spontaneous groundcovers were regarded as more natural, wild, variable, and species-richer compared to lawns, while lawns were perceived as better kept than spontaneous groundcovers. Ground covers were preferred which were perceived to have high ecological aesthetic value and low wildness. Industry and attention to herbaceous plants mostly affected human perceptions and preferences among the social background factors, and gender, age, education level, and occupation also had significant effects. The results thus provide the support for the application of spontaneous groundcovers in moderately developed cities, but such application should consider the comprehensive development of ecological aesthetic value and the applicability of different groups of residents.

## 1. Introduction

Historically, lawns have stood as prominent symbols in cities globally (Ignatieva and Hedblom, 2018). Lawn is fine-textured turf of grass that is kept mowed (The Editors of Encyclopaedia Britannica, 2013), originally composed of a mixture wild grasses and mowing tolerant wildflowers native to the relatively moist and mild maritime climate of Northwest Europe (Smith and Fellowes, 2015). In China, the term “lawn” encompasses a broader connotation that it is a surface made up of herbaceous plants, established and managed artificially and offering both aesthetic and recreational values (Xia and Zhao, 2000). Lawns provide positive ecosystem services like reducing runoff, increasing infiltration, purifying water from sediments and pollutants, controlling erosion, improving soil quality and providing wildlife habitat (Monteiro, 2017). Notably, the visual appeal of lawns underscores their importance, with many cultures valuing the neatness and order they bring (Monteiro, 2017; Ignatieva and Hedblom, 2018).

However, the overall use of lawns has caused concerns. Lawns worsen problems brought on by population increase and climate change, such as rising greenhouse gas emissions and declining urban biodiversity (Ignatieva and Hedblom, 2018). Lawn irrigation consumes a huge amount of water every year (Milesi et al., 2005). The frequent use of fertilizer and pesticide due to intensive management causes environment pollution – lawn chemicals have been found in 99% of urban rainwater samples in the United States (Cheng et al., 2008; Alumai et al., 2009). In addition, the positive effect of soil carbon sequestration on the climate footprint of intensively managed lawns was found to be negated by greenhouse gas emissions from management operations such as mowing, irrigation, and fertilization (Tidåker et al., 2017). From an aesthetic perspective, tidily trimmed lawns are not always familiar to residents in different geographical contexts. While prevalent in western countries, lawns were historically absent from traditional Chinese gardens, their current ubiquity stemming from Western influences (Yang et al., 2019a). Consequently, research has pivoted toward exploring lawn alternatives like grass-free lawns, flowering lawns, and urban meadows that offer advantages in maintenance, aesthetics, and biodiversity (Smith and Fellowes, 2015; Ramer et al., 2019).

Urban spontaneous vegetation refers to the vegetation that grows naturally and spontaneously in urban sites (Li et al., 2019b). Normally, it was not intentionally planted and cultivated by humans (Cervelli et al., 2013) and was often referred to as “weeds,” which are indicators of mess and are thus removed from parks and gardens (Li et al., 2019b). As people have begun to pay attention to sustainable alternatives to urban lawns, the spontaneous vegetation has been gradually noticed due to its obvious benefits including supporting biodiversity (Villasenor et al., 2020), increasing carbon sequestration and organic carbon content (Chamizo et al., 2017), restoration of the soil environment (Boechat et al., 2016), etc.

While the ecological superiority of spontaneous vegetation is evident, its acceptability as a lawn substitute among urban dwellers remains ambiguous. Studies in Sweden, Singapore and the United Kingdom showed city dwellers appreciated natural diverse grassland and wild flowerbeds over monotonous lawns (Ignatieva, 2017; Southon et al., 2017; Hwang et al., 2019). Yet still, the wild look of spontaneous vegetation is often regarded as messy, dangerous and brings about the thought of a place being abandoned (Hands and Brown, 2002; Lyytimäki et al., 2008), potentially deterring its adoption. Li et al. (2019b) found that Chinese residents favored short-cut lawns and traditional flowerbeds, while spontaneous vegetation with specific features was only preferred by people with more exposure to nature and higher levels of education, and who are gardeners, landscape architects, and professional students. Given the divergent findings across various studies and contexts, there is an imperative for systematic comparisons to derive meaningful conclusions for urban design.

The public’s perception of lawns and spontaneous groundcovers may be the motivation of human preference and utilization of such. Yang et al. (2019b) found lawns were valued for their ecological and aesthetical values but least appreciated as important for recreational activities in Xi’an, China. The respondents in a similar study (Teixeira et al., 2022) realized the benefits of spontaneous plants in resisting climate change and enriching biodiversity, but also showed desire for some care and maintenance, which led to their preference for combinations of cultivated and spontaneous plants. Social background including gender, age, education level, occupation, living environment, income, and especially the possession of ecological knowledge, were also proved to have effects on urban ground cover perception and preference (Lindemann-Matthies et al., 2010; Jiang and Yuan, 2017; Fischer et al., 2018; Li et al., 2019a). Thus, to understand people’s preference, it is necessary to analyze individual perceptions and social background factors.

A series of case studies have centered on developed areas such as Europe and North America, and highly urbanized cities in China like Beijing and Xi’an (e.g. Mathey et al., 2018; Li et al., 2019b; Yang et al., 2019b; Phillips and Lindquist, 2021). Many underdeveloped Chinese cities such as Xianyang with medium to low economic and education levels, have prominent contradictions between socio-economic growth and ecological conditions (Shi et al., 2020; Xiao et al., 2022), and therefore should also be considered. In addition, these cities are facing intense environmental changes including habitat fragmentation and the increase of non-native plants due to rapid urban expansion and large population growth (Zhao et al., 2010), with the additional burden of increasing recreational needs of urban residents at the same time. With these circumstances in mind, this study investigated people’s perceptions and preferences regarding the common spontaneous groundcovers and lawns in Xianyang, China, and explored the social background factors that affect people’s attitudes in order to inform urban design strategies. Since the definition of lawn in China has not been unified (Yang et al., 2019a) and usually includes other cultivated groundcovers, the lawn in this study mainly includes conventional short-cut lawn and a few common monoculture groundcover that artificially planted in the local area. The spontaneous groundcover refers to the low spontaneous plant community that can serve as a groundcover in green spaces. This study aims to address the following questions:

(1) How do the residents perceive spontaneous groundcovers and lawns?
(2) How do the residents like spontaneous groundcovers and lawns (what are their preferences)?
(3) What is the relationship between people’s perceptions of and preferences for different ground covers?
(4) To what extent do people’s social background factors affect perceptions of and preferences for different ground covers?

## 2. Materials and methods

### 2.1. Study site and ground cover selection

The study site was conducted in Xianyang city, China, characterized by a warm temperate zone in East Asia with a semi-humid and semi-arid continental monsoon climate. It is cold and dry in winter with temperatures dipping to around −6°C in January, and hot and rainy in summer, peaking at around 32°C in July. By 2020, the per capita GDP of Xianyang city was USD 7,665.75, below the national average of USD 10,000.80. Its urbanization rate (proportion of urban population to total population) was 55.44%, which is also lower than the national average of 63.89% (Xianyang Statistics Bureau, 2021). As in most Chinese cities, lawns in Xianyang became popular at the end of the 20th century and the beginning of the 21st century (Yang et al., 2019a). Largely due to the nation’s garden city construction program and the region’s climate constraints, the lawns (mostly cool-season lawns) covered a large area of urban green space.

A pre-investigation spanning 2019–2021 was conducted for ground cover selection, focusing on five types of urban green spaces (park, protective green space, plaza, attached green space, and regional green space) in Xianyang according to the latest national standard for classification of urban green space in China (CJJ/T85-2017) (Ministry of Housing and Urban-Rural Development of the People’s Republic of China, 2018). Additional field surveys extended to surrounding rural areas, wastelands and Qinling mountain, a vital place of surrounding plant resources. The lawns appeared in all the five types of urban green spaces, and the spontaneous groundcovers were usually distributed in informal urban green spaces, abandoned grasslands, and urban-rural junctions. Consultations with local seedling companies and lawn experts further informed our selection. Finally, six kinds of spontaneous groundcover communities were selected which appeared frequently and grew stably, and seven kinds of lawns were also confirmed which were used most commonly in urban green spaces in the semi-humid and semi-arid climate zone (Table 1).

**TABLE 1:** Types of ground covers in the field.

Type	Kind	Abbreviation of name	Planting process
Spontaneous groundcover	*Trigonotis peduncularis – Cynodon dactylon – Bromus catharticus*	TCB	Transplanting
	*Gueldenstaedtia verna – Cynodon dactylon – Cerastium glomeratum*	GCC	Transplanting
	*Medicago lupulina – Cynodon dactylon – Capsella bursa-pastoris*	MCC	Transplanting
	*Coronilla varia – Veronica persica – Cynodon dactylon*	CVC	Transplanting
	*Taraxacum mongolicum – Cynodon dactylon – Conyza canadensis*	TCC	Transplanting
	*Duchesnea indica*	D	Transplanting
Lawn	*Cynodon dactylon* × *C. transvaalensis*	C	Turfing
	*Poa pratensis*	P	Turfing
	*Oxalis corymbosa*	O	Planting
	*Festuca elata – Poa pratensis*	FP	Turfing
	*Festuca elata – Poa pratensis – Lolium perenne*	FPL	Turfing
	*Trifolium repens*	T	Planting
	*Dianthus deltoides × hybrida*	DH	Planting

The groundcover names are displayed in order of dominant species.

**FIGURE 1:**
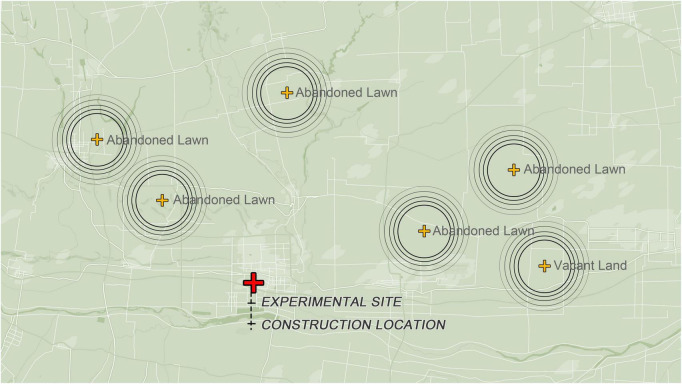
The distribution of initial habitats of the spontaneous groundcovers.

In order to avoid the participants’ perceptions and preferences being influenced by varying contexts, all ground covers were planted in 39 planting units (2 m × 2 m each) on the study site with every kind of ground cover repeatedly planted in three units in May 2021. The lawns were constructed by turf or seedlings bought from the local nursery. The spontaneous groundcovers were transplanted from initial habitats, which were nearby vacant lands and abandoned lawns, to every unit (Figure 1). The underground part of the selected spontaneous groundcover communities was cut into squares with a length of 40 cm, a width of 20 cm and a depth of 30 cm, and transplanted and spliced in the sample units. All the spontaneous groundcovers were combinations of native and non-native plants. As for the lawns, the *Oxalis corymbosa* lawn and *Poa pratensis* lawn were both native plants, and the other lawns were composed of non-native plants.

### 2.2. Ground cover maintenance and observation

Proper maintenance is pivotal for both lawn and spontaneous ground covers to ensure health growth and prevent excessive flourishing in urban landscape. According to the watering principle adopted by Lilly et al. (2015), combined with the empirical value of local lawn watering quantity, a best practice method was chosen which was to irrigate every unit twice a week, 1.5 cm a time, including any natural rainfall. This method has been acknowledged in several studies (e.g. Taylor, 1998; Goatly, 2009). The spontaneous groundcovers were only watered when the blades were yellow or wilting, and the water amount was 3 cm each unit at a time.

In order to maintain adequate ornamental characteristics that are normal for ground covers and encourage them to grow well, the mowing height of spontaneous groundcovers and lawns was determined according to the experiences of local daily management coupled with the mowing method adopted by Lilly et al. (2015). The plots were checked weekly in the growing season and mowing was conducted once the vegetation attained a predetermined height. Spontaneous groundcovers were cut to 15 cm once they reached a height of 20 cm. The lawns were cut to different heights, based upon kind: T was cut to 12 cm at 18 cm; C was cut to 5 cm at 8 cm; FPL was cut to 10 cm at 15 cm and P was cut to 8 cm at 12 cm. Lawn kinds O and DH were not mowed because their heights did not exceed 8 cm across all growing seasons. Every 10 days, the seedlings of woody plants and lianas found in the experimental plots were removed to avoid their interference to the normal succession of the groundcover communities. During the experiment, some vines were found in the spontaneous communities, such as *Calystegia hederacea*, *Humulus scandens*, and *Cayratia japonica*, along with some woody seedlings such as *Ligustrum lucidum* and *Triadica sebifera*. For lawns, all plants except for the lawn itself were removed. All of the planting, management, and maintenance was completed by professional gardeners.

In order to understand the growth and development of the ground covers over time, the number of plant species in every ground cover community was observed and calculated, based on which the dominant species was also determined (Table 2). The species in the lawns were maintained as consistent with the original planting.

**TABLE 2:** The plant species in ground covers in winter and spring.

Type	Kind	Plant species in winter	Plant species in spring
Spontaneous groundcover	TCB	***Cynodon dactylon*** *, Artemisia lavandulaefolia, Bromus japonicus, Geranium wilfordii, Ixeris polycephala, Veronica persica*	***Cynodon dactylon*** *, Artemisia lavandulaefolia, Bromus japonicus, Veronica persica, Cerastium glomeratum, Trigonotis peduncularis, Capsella bursa-pastoris, Sonchus oleraceus, Sonchus asper*
	GCC	***Veronica persica***, *Cynodon dactylon, Artemisia lavandulaefolia, Gueldenstaedtia verna, Geranium wilfordii, Carex tristachya, Ixeris polycephala, Trifolium repens, Rumex acetosa, Taraxacum mongolicum*	***Cynodon dactylon*** *, Artemisia lavandulaefolia, Veronica persica, Gueldenstaedtia verna, Trifolium repens, Cerastium glomeratum, Medicago lupulina, Taraxacum mongolicum, Rumex acetosa*
	MCC	***Cynodon dactylon*** *, Ixeris polycephala, Veronica persica, Medicago lupulina, Sonchus oleraceus, Rumex acetosa, Oxalis corymbosa, Althaea rosea*	***Medicago lupulina*** *, Capsella bursa-pastoris, Cynodon dactylon, Veronica persica, Sonchus oleraceus, Oxalis corymbosa, Rumex acetosa, Althaea rosea, Ixeris polycephala, Bromus japonicus, Stellaria media, Euphorbia helioscopia*
	CVC	***Coronilla varia*** *, Veronica persica, Bromus japonicus*	***Veronica persica*** *, Coronilla varia, Capsella bursa-pastoris, Sonchus asper, Bromus japonicus, Geranium wilfordii, Cerastium glomeratum*
	TCC	***Cynodon dactylon*** *, Coronilla varia, Poa pratensis, Veronica persica, Duchesnea indica*	***Cynodon dactylon*** *, Coronilla varia, Poa pratensis, Veronica persica, Capsella bursa-pastoris, Carex tristachya, Cerastium glomeratum, Ixeris polycephala, Lolium perenne, Viola philippica, Inula japonica, Oxalis corymbosa, Taraxacum mongolicum, Erigeron annuus*
	D	***Duchesnea indica*** *, Inula japonica, Digitaria sanguinalis*	***Duchesnea indica*** *, Inula japonica, Poa pratensis, Veronica persica, Euphorbia helioscopia*
Lawn	C	***Cynodon dactylon × C. transvaalensis***	***Cynodon dactylon × C. transvaalensis***
	P	***Poa pratensis***	***Poa pratensis***
	O	***Oxalis corymbosa***	***Oxalis corymbosa***
	FP	***Festuca elata*** *, Poa pratensis*	***Festuca elata*** *, Poa pratensis*
	FPL	***Festuca elata*** *, Poa pratensis, Lolium perenne*	***Festuca elata*** *, Poa pratensis, Lolium perenne*
	T	***Trifolium repens***	***Trifolium repens***
	DH	***Dianthus deltoides × hybrida***	***Dianthus deltoides × hybrida***

Bold words indicate dominant species.

### 2.3. Photograph and questionnaire

**FIGURE 2:**
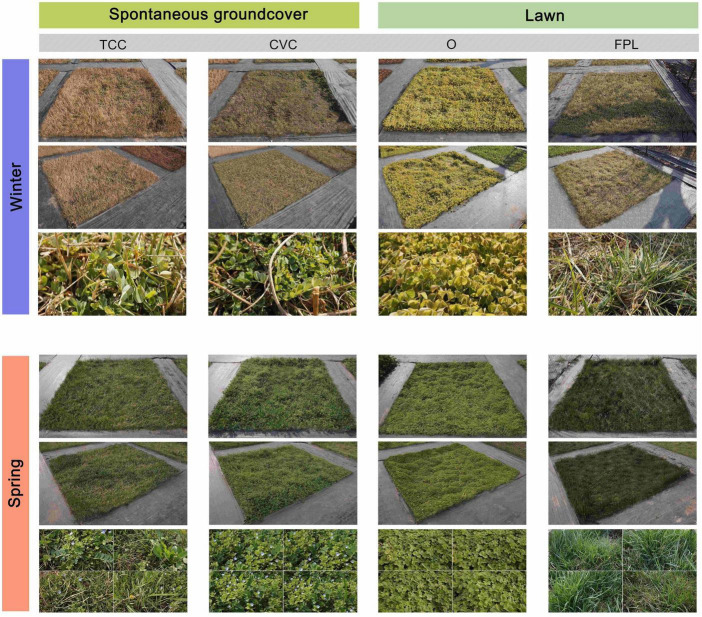
Examples of photo materials of ground covers taken from three angles.

In December 2021 and March 2022, three unaltered photographs of each kind of ground cover were taken. The first and second photographs were taken at a height of 1.5 m, from the parallel perspective and angulation perspective, respectively. The third photograph was taken at a height of 0.3 m, representing the details of the communities (Figure 2). Since the ground covers varied in spring, four detailed photos were taken to show the varying appearances of the communities and were then compiled into one photograph in March. The photographs were printed in high resolution at the size of 297 mm × 180 mm for use in subsequent interviews.

The questionnaire consisted of two parts. The first part focused on residents’ perceptions of and preference for the ground covers showcased in the photos. Drawing from established literature on ground cover perceptions (Gyllin and Grahn, 2005; Ode et al., 2009; Brun et al., 2018), eight pairs of antithetical semantic attributes to evaluate the perceptions of ground covers were incorporated, including “artificial/natural,” “domesticated/wild,” “boring/interesting,” “ugly/beautiful,” “neglected/well kept,” “monotonous/varied,” “non-ecological/ecological,” and “with few species/with rich species.” Participants were asked to rate their perceptions and preferences using five-point Likert scales. For example, for attributes “artificial/natural”, 1 = very artificial, 2 = a little artificial, 3 = neutral, 4 = a little natural, 5 = very natural. Preference was assessed through the question “How do you like these photos?” on a continuous five-point scale (1 = very dislike, 5 = very like). Participants were probed on the reasons behind the choices. The second part of the questionnaire contained the personal information of the participants, including gender, age, education level, occupation, monthly income, industry, living environment, and attention to herbaceous plants. Participants’ attention to herbaceous plants were assessed through the question “Have you ever paid attention to the herbaceous plants around you in your everyday life?”

### 2.4. Survey process

The surveys were conducted in Xianyang in December, 2021 and March, 2022. People in public outdoor spaces like parks, plazas, and roadsides were randomly invited to participate in the survey. Each participant was asked to look at the three photos of one kind of ground cover and complete the questionnaire. An initial total of 4,150 people were recruited for the survey and 12 were excluded due to incomplete questionnaires. Finally, a total of 4,138 participants were included.

### 2.5. Data analysis

All statistical analyses were carried out using SPSS 20.0 software. The two sample *T*-test and one-way ANOVA were used to explore the differences in residents’ perceptions of and preferences for spontaneous ground covers and lawns, and the 13 kinds of ground covers. A principal component analysis (PCA) was executed to extract prominent factors for the semantic attributes of perception. A multiple linear regression analysis was conducted to explore the effects of perceptions on preferences. To explore the effects of social backgrounds on perceptions and preferences, a generalized liner model was used. The significant level used was 0.05 in this study.

## 3. Results

**FIGURE 3:**
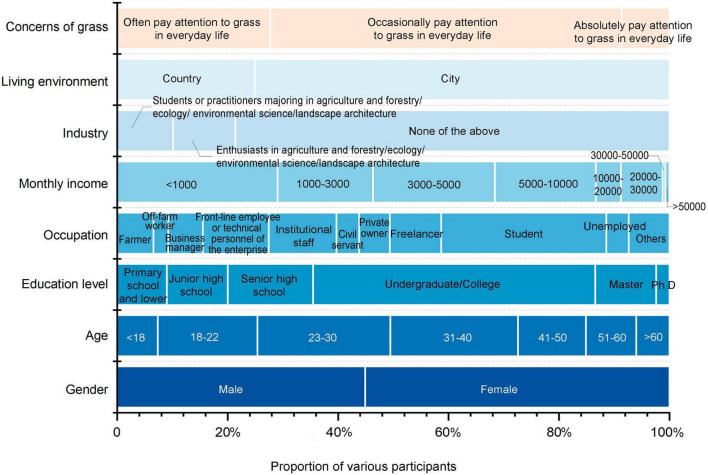
Social background characteristics of the participants.

Out of all the participants, 1,858 (44.90%) were male. The age of participants was mostly distributed across the range of 18–40 (47.17%). Most participants (51.09%) held a bachelor’s degree or other college degree. Students occupied the largest number across all occupations (29.89%), spanning primary to tertiary education levels. A considerable majority (78.64%) were not affiliated with industries related to agriculture, forestry, ecology, or landscape architecture, and quite a few participants (63.65%) said that they only paid attention to herbaceous plants occasionally in their lives (Figure 3 and Supplementary Table 1).

### 3.1. Residents’ perceptions of spontaneous groundcovers and lawns

Residents’ perceptions of spontaneous groundcovers and lawns were found to have significant differences in five dimensions (Table 3): artificial/natural (*p* < 0.01), domesticated/wild (*p* < 0.01), neglected/well kept (*p* < 0.01), monotonous/varied (*p* < 0.01) and with few species/with rich species (*p* < 0.01). Spontaneous groundcovers were regarded as more natural, wild, varied, and species-richer than lawns, whereas lawns were viewed as better kept than spontaneous groundcovers. There was no significant difference in the categorizations of boring/interesting, ugly/beautiful, or non-ecological/ecological.

**TABLE 3:** Perceptions of spontaneous groundcovers and lawns.

	*F*	*t*	df	Sig.
Artificial/natural	6.30	5.02	4,133.78	**<0.01**
Domesticated/wild	0.15	8.78	4,136.00	**<0.01**
Boring/interesting	4.98	−0.23	4,134.58	0.82
Ugly/beautiful	7.02	−1.89	4,135.99	0.06
Neglected/well kept	0.15	−6.80	4,136.00	**0.01**
Monotonous/varied	0.28	2.58	4,136.00	**0.01**
Non-ecological/ecological	5.35	0.05	4,135.30	0.96
With few species/with rich species	2.45	6.91	4,136.00	**<0.01**

Bold type indicates significant differences.

### 3.2. Residents’ preferences for spontaneous groundcovers and lawns

**FIGURE 4:**
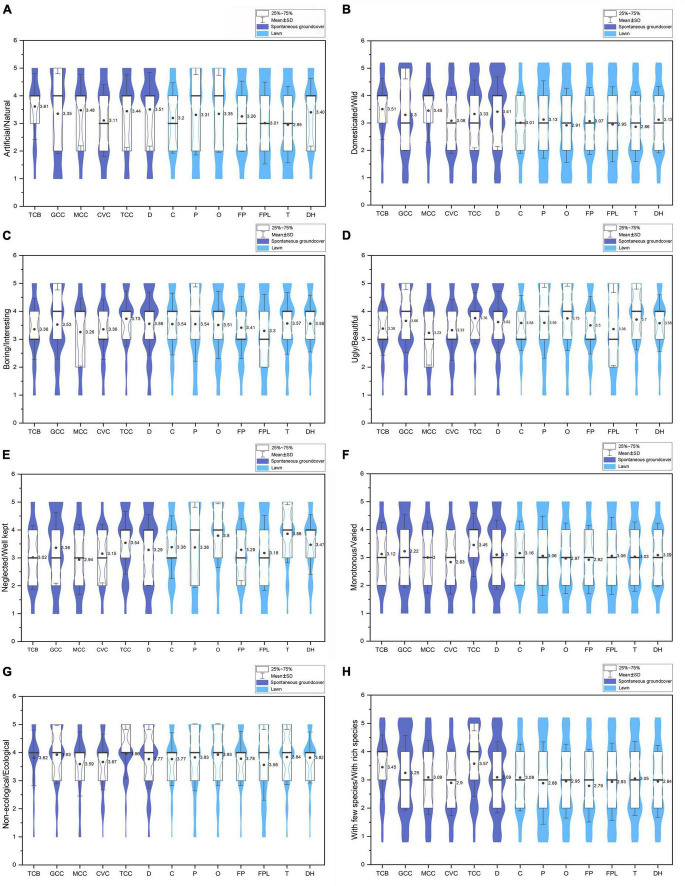
Perceptions of 13 ground covers about eight attributes. **(A)** artificial/natural, **(B)** domestic/wild, **(C)** boring/interesting, **(D)** ugly/beautiful, **(E)** neglected/well kept, **(F)** monotonous/varied, **(G)** non-ecological/ecological, and **(H)** with few species/with rich species.

The result of the one-way ANOVA indicated that residents’ perceptions were significantly different among the 13 ground cover sites (Table 4). Spontaneous groundcover kind *T. mongolicum – C. dactylon – C. canadensis* (TCC) received the highest scores in almost all of the perception attributes (Figure 4). Kinds *O. corymbosa* (O) and *Trifolium repens* (T) also earned high scores among lawns in attributes except for domesticated/wild, monotonous/varied, and with few species/with rich species. FPL received fairly low scores in most attributes and MCC received lower scores than other spontaneous groundcovers except for perceptions of artificial/natural and domesticated/wild attributes. Generally, among all the attributes, people gave the highest scores to perceptions of non-ecological/ecological, followed by ugly/beautiful and boring/interesting, while the mean value of perceptions of monotonous/varied, with few species/with rich species, and domesticated/wild were much lower than other attributes (Figure 4).

**TABLE 4:** The effects of the groundcover types on eight perception attributes through one-way ANOVA.

Attributes	Sum of squares	Mean square	*F*	Sig.
Artificial/natural	145.890	12.158	6.759	**0.000**
Domesticated/wild	176.187	14.682	9.270	**0.000**
Boring/interesting	65.773	5.481	4.144	**0.000**
Ugly/beautiful	105.163	8.764	7.305	**0.000**
Neglected/well kept	274.845	22.904	16.152	**0.000**
Monotonous/varied	90.808	7.567	4.923	**0.000**
Non-ecological/ecological	54.605	4.550	4.247	**0.000**
With few species/with rich species	196.984	16.145	10.105	**0.000**

Bold values indicate significant differences.

**FIGURE 5:**
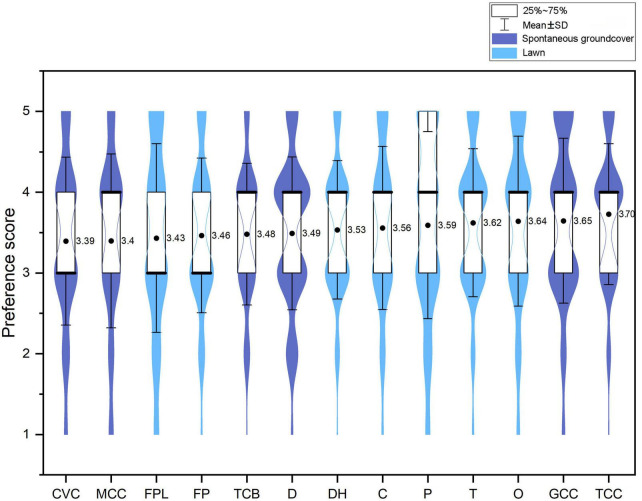
Preference scores for 13 ground covers. Groups with different letters were significantly different.

The result of the *T*-test showed that there was no significant difference in residents’ preferences between spontaneous groundcovers and lawns (*t* = 0.616, *p* = 0.538). The result of a one-way ANOVA showed that there was a significant difference in preferences among the 13 sites (*F* = 3.217, *p* < 0.01). TCC received the highest preference score, followed by GCC, O, T, P, C, DH, D, and TCB. Specifically, the score for TCC was significantly higher than those measured for CVC, MCC, FPL, and FP (Figure 5).

### 3.3. The relationship between perceptions of and preferences for ground covers

**FIGURE 6:**
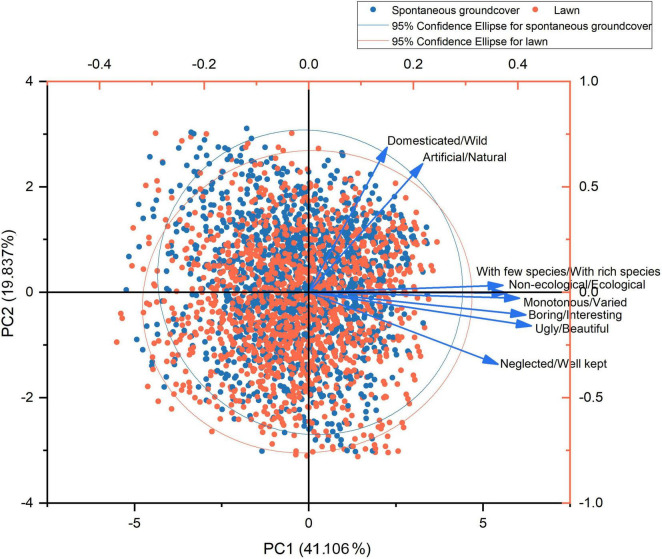
The variance of eight pairs of attributes explained by component 1 and component 2 through PCA.

The results of the Kaiser–Meyer–Olkin test (KMO = 0.831) and Bartlett’s Test of Sphericity (*p* = 0.000) showed that PCA could be used to extract prominent dimensions for the perception-related semantic attributes. The explained variance of each component is reported in Supplementary Table 2 and the first two components were retained, with total explanatory power of 60.942%. Component 1 exhibited 41.106% explanatory power and included “boring/interesting,” “ugly/beautiful,” “monotonous/varied,” “non-ecological/ecological,” “with few species/with rich species,” and “neglected/well kept,” and was therefore labeled ecological aesthetics. Component 2 exhibited 19.837% explanatory power and included “artificial/natural” and “domesticated/wild,” and was therefore labeled wildness (Figure 6).

Of the two components used in the multiple linear regression, the results showed that perception on ecological aesthetics had a significant positive effect on preference and the effect was relatively strong (*p* = 0.000, *B* = 0.679). The perception on wildness had a significant negative effect, although the effect was weak (*p* = 0.000, *B* = −0.115) (Table 5).

**TABLE 5:** The effects of perceptions on preferences for ground cover through multiple linear regression.

Model	*B*	SE	*t*	Sig.	Tolerance	VIF
(Constant)	3.543	0.011	313.963	0.000		
Ecological aesthetics	0.679	0.011	60.133	**0.000**	1.000	1.000
Wildness	−0.115	0.011	−10.222	**0.000**	1.000	1.000

Bold type indicates significant differences (adjusted *R*^2^ = 0.473).

### 3.4. The effect of social background on perceptions of and preferences for ground covers

The results of the generalized liner model showed that education level, industry and attention to herbaceous plants had significant effects on ecological aesthetics, while gender, age, occupation, monthly income, and living environment had no significant effect (Table 6). People who had obtained a master’s degree, who had engaged in ecology-related industries and those who hardly paid attention to herbaceous plants in daily life tended to appreciate less ecological aesthetics (Supplementary Table 3). Gender, age, occupation, industry, and attention to herbaceous plants had significant effects on perceived wildness, while education level, monthly income, and living environment had no significant effect (Table 6). Women, people under 18 years old and farmers perceived a significantly higher degree of wildness than other participant groups in general. Men, the elderly, enthusiasts about ecology and people who often paid attention to herbaceous plants showed lower perceptions of wildness (Supplementary Table 4).

**TABLE 6:** The effects of social background on perceptions of and preference for ground covers through a generalized linear model.

Origin	Ecological aesthetics			Wildness			Preference		
	**Wald χ^2^**	**df**	**Sig.**	**Wald χ^2^**	**df**	**Sig.**	**Wald χ^2^**	**df**	**Sig.**
(Intercept)	4.303	1	0.038	2.291	1	0.130	4415.419	1	0.000
Gender	0.023	1	0.879	16.203	1	**0.000**	6.523	1	**0.011**
Age	10.663	6	0.099	19.503	6	**0.003**	12.924	7	**0.044**
Education level	11.372	5	**0.044**	7.365	5	0.195	17.333	5	**0.004**
Occupation	16.786	10	0.079	22.670	10	**0.012**	18.361	10	**0.049**
Monthly income	7.111	7	0.417	11.115	7	0.134	9.984	7	0.190
Industry	11.695	2	**0.003**	6.520	2	**0.038**	17.541	2	**0.000**
Living environment	0.248	1	0.619	0.867	1	0.352	0.189	1	0.664
Attention to herbaceous plants	124.273	2	**0.000**	9.302	2	**0.010**	133.409	2	**0.000**
Ground cover kind	83.436	12	**0.000**	182.092	12	**0.000**	37.767	12	**0.000**

Bold type indicates significant differences.

**FIGURE 7:**
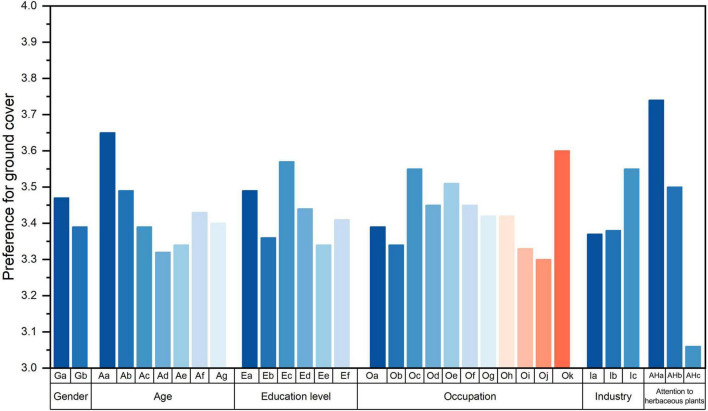
Participants’ preferences with different social backgrounds. Only social backgrounds with significant differences are shown. Detailed information concerning the model can be seen in Supplementary Table 5. The variables represented by the letters are: Ga – male, Gb – female; Aa – below 18, Ab – 18∼22, Ac – 23∼30, Ad – 31∼40, Ae – 41∼50, Af – 51∼60, Ag – over 60; Ea – primary school and lower, Eb – junior high school, Ec – senior high school, Ed – undergraduate/college, Ee – master, Ef – Ph.D.; Oa – farmer, Ob – off-farm worker, Oc – business manager, Od – front-line employee or technical personnel of the enterprise, Oe – institutional staff, Of – civil servant, Og – private owner, Oh – freelancer, Oi – student, Oj – unemployed, Ok – others; Ia – students or practitioners majoring in agriculture and forestry/ecology/environmental science/landscape architecture, Ib – enthusiasts in agriculture and forestry/ecology/environmental science/landscape architecture, Ic – none of the above; Aha – often pays attention to herbaceous plants in everyday life, Ahb – occasionally pays attention to herbaceous plants in everyday life, Ahc – absolutely pays attention to herbaceous plants in everyday life.

As for preference for ground covers, gender, age, education level, occupation, industry, and attention to herbaceous plants had significant effects on preference, while monthly income and living environment had no significant effect (Table 6). People with senior high school education, those engaged in other occupations (mostly housewives), and participants who often paid attention to herbaceous plants showed significantly higher preferences. People with junior high school education or with a master’s degree, off-farm workers, unemployed participants, students, and practitioners majoring in agriculture and forestry/ecology/environmental science/landscape architecture and people who never paid attention to herbaceous plants were found to have significantly lower preferences than others (Figure 7 and Supplementary Table 5).

## 4. Discussion

### 4.1. How do the residents perceive spontaneous groundcovers and lawns?

Compared to lawns, the participants discerned spontaneous groundcovers as more natural, wilder, more neglected, more varied and with richer species, indicating residents’ perceptions were in correspondence with reality. Given the pervasive presence of urban short-cut lawns throughout the region, residents are well-acquainted with them and can readily differentiate them from spontaneous groundcovers based on their wild appearance and maintenance standards, especially lawns which were trimmed neatly and mainly composed of grasses.

Interestingly, there were no significant differences in terms of the aesthetic feature, attraction, and ecological traits between the two types of ground covers. Although there is evidence that higher naturalness contributes to higher aesthetic values in urban green spaces (Ode Sang et al., 2016), excessive wildness and messy appearances of ground covers will also reduce aesthetic perceptions. Prior studies have showed the aesthetic appreciation was related to vegetation structure (Wang et al., 2017) and colors (Hoyle et al., 2018) in urban green spaces. In this study, given that the showcased ground covers are local, possess homogenous structures, and lack pronounced color differences, the ground covers seemed to be aesthetically similar.

Participants’ perceptions of the ecology feature were also much the same, with the ecology feature receiving the highest scores across perception metrics. During the survey, most people thought that compared with the built area, all plants inherently bolster the environment, thus classifying them as “ecological.” Clergeau et al. (2001) found that the city residents often lacked the experience and skills necessary to identify plants and appreciate biodiversity. Therefore, they tended to think the ground covers were ecologically beneficial without distinguish their differences.

### 4.2. How do the residents like spontaneous groundcovers and lawns (what are their preferences)?

Participants exhibited comparable preferences for both spontaneous groundcovers and traditional lawns, which shows that people’s acceptance of native ground cover parallels that of lawn. After the housing reform and the “National Garden City” program in the 1990s, the 2008 Beijing Olympic Games and other construction projects, the residential areas and urban public green spaces in China were quickly covered by lawns. These lawns, while offering rapid urban greening, also symbolize a western lifestyle, which, given China’s economic trajectory at the time, held significant appeal (Yang et al., 2019a). However, as urbanization has proliferated, the once-unique allure of lawns has waned, replaced by an increasing societal inclination toward natural settings. Research in China has underscored a preference for natural grasslands over traditional manicured lawns (Jiang and Yuan, 2017), and the intrinsic value of spontaneous vegetation has gained recognition among professionals (Li et al., 2019b; Yang et al., 2019b). Given the economic and urbanization level of Xianyang, which sit below the national averages, there may be no obvious difference in residents’ preference for spontaneous groundcovers and lawns. However, as the city evolves, the widespread adoption of lawns may not align with evolving resident needs, positioning spontaneous groundcovers as a potentially popular alternative.

The most popular spontaneous groundcover was the *T. mongolicum* – *C. dactylon – C. canadensis* community, which received the highest perceptions across almost all the attributes. Oppositely, the spontaneous groundcovers *Coronilla varia* – *Veronica persica* – *C. dactylon* and *Medicago lupulina* – *C. dactylon* – *Capsella bursa-pastoris*, and the lawns *Festuca elata – P. pratensis – Lolium perenne* and *F. elata – P. pratensis* received the lowest preferences and also earned relatively lower perceptions across most attributes. These findings mean the preference for ground cover type is not absolute and instead is related to the performance of specific communities. Spontaneous groundcover kind TCC was composed of richer species and was perceived as being ecological, natural, beautiful and well kept, while kinds CVC and MCC, also spontaneous groundcovers, contained fewer species and were perceived as being more neglected, monotonous, ugly and boring. The differences among the spontaneous groundcovers led to the differences in terms of coherence and complexity of landscape. In Kaplan and Kaplan’s (1989) landscape preference matrix, the term coherence represents an orderly and organized setting with limited and repeating themes, and complexity represents the richness of a setting. Kuper (2017) found that landscape preference did correlate with evaluations of complexity. There were abundant species with different morphological features (e.g., leaf shape, flower shape, and color) in TCC in spring (Figure 2), with the highest score measured in species richness perception. Species diversity also proved to be related to human preference (Lindemann-Matthies et al., 2010; Southon et al., 2017). By contrast, FPL and FP, both composed of grass, showed highly homogeneous looks and low complexity, and thus were disliked.

Although there were also many plant species found in kinds CVC and MCC, the plant morphology found within them showed a bad order, especially in winter. For instance, the leaves of *C. varia*, the dominant species of CVC, fall off in winter, leaving messy yellow stems (Figure 2). Zhang et al. (2021) reported people prefer a landscape with high richness when the landscape keeps a good sense of order, which is one aspect of coherence. In addition, order to some extent represents “being cared for” in landscapes, and thus is perceived in landscapes which are valued (Nassauer, 2011).

Generally, results indicate that the spontaneous groundcovers have the potential to replace or even surpass the lawns in respect to gaining people’s appreciation due to the richness of their components, but the groundcover kind should be selected cautiously to exhibit good order. These findings provide solid evidence in favor of rewilding practices. Rewilding, which originated in Germany and was primarily focused on wilderness areas like national parks and nature reserves, has since evolved into a new technique for ecological restoration that aims to minimize human intervention, emphasize the importance of natural restoration in the process and mechanism of ecosystem self-maintenance and self-regulation, place a strong emphasis on extensive native species restoration, appropriately reintroduce key species of ecosystems, and restore ecosystem function (Lorimer et al., 2015; Kowarik, 2018). As products and important tools in the process of rewilding, the spontaneous plants have evolved into a practical design strategy that enhances urban landscapes (Kühn, 2006), used in worldwide projects like Jiang Yang Fan Eco-Park in China, Landscape Park Duisburg-Nord in Germany, Carl-Alexander Park in the United States, and High Line Park in New York.

### 4.3. What is the relationship between people’s perceptions of and preferences for different ground covers?

The results indicate that people tended to prefer ecological, good-looking, and less wild ground covers. The term “ecology” resonated positively among participants, often being equated with environmental benefits. As such, there was a discernible inclination toward ground covers perceived as ecologically vibrant and species-rich.

Yet, people still attached importance to the aesthetic value, which is not always consistent with ecological quality (Gobster et al., 2007). Although people showed great interest in approaching more natural and biologically rich environments (Carrus et al., 2015; Hwang et al., 2019), they value the order and neatness over pure wildness. Wildness often conjures images of disorder, potentially invoking feelings of unease or perceived danger (Nassauer, 1995; Mathey et al., 2018; Li et al., 2019b). Therefore, the positive ecological aesthetic preference can be seen as an expectation for urban ground covers but not reflective of the reality for the quality of ground covers. In order to maintain adequate ecological and aesthetical values and avoid wildness, the application of the spontaneous groundcover requires the introduction of human stewardship, which was termed as “cues to care” (Nassauer, 1995, 2011), into daily management. Moreover, enhanced ecological knowledge might change the public’s response to the groundcovers (Gobster et al., 2007) and should be introduced more to help glean greater information about ground cover preference.

In general, the preference for ground covers is a composite reflection of considering various perceptions of attributes which results in the balance of ecological and aesthetic values. The results imply that ground cover, with no distinction for lawns or spontaneous groundcovers, with attractive appeal, better ecological functions and a touch of human stewardship, should be adopted in urban green spaces.

### 4.4. To what extent did people’s social background factors affect perceptions of and preferences for different ground covers?

Professional experience and attention to herbaceous plants emerged as significant determinants of ground cover perceptions among the demographic characteristics. Previous studies in multi-scales showed that differences appeared in how green experts vs. laymen assess urban greenery (Muratet et al., 2015; Fischer et al., 2018). The results of the present study indicated that people who were engaged in ecology-related industries tended to appreciate less ecological aesthetics, but still showed a higher preference for them. Li et al. (2019b) reported that professionals could better recognize and preferred the value of spontaneous vegetation than traditional plantings. In this study, due to the experimental design that each participant only assessed one kind of ground cover, the ground cover seemed to be identified as less functional and less aesthetically pleasing, because diverse plant communities containing rich plant diversity and diverse vegetation structures are generally preferred (Qiu et al., 2013; Giergiczny et al., 2015; Gao et al., 2019). The results also showed that ecology enthusiasts showed lower perceptions of wilderness. Chances are that these people have enough knowledge and experience in observing nature and plants, but are short of professional knowledge, leading to their tolerance to wilder groundcovers but low consciousness of wilderness. Nevertheless, the present study expands existing knowledge in the comparison between experts and laymen, showing the similar preference results as found in previous studies, but revealing differences in perception under a pure single evaluation.

It has been shown that when residents have stronger ties to nature, they value plant species richness more (Lindemann-Matthies and Bose, 2007). In this study, people who usually paid attention to herbaceous plants in daily life recognized higher ecological aesthetics and wildness value, and also held higher preferences for them. Results of the questionnaire indicated this group was easily pleased with all kinds of ground covers, because they provided greenery and increased the chances for people to get close to nature, no matter which kinds they were.

Gender, age, education level and occupation were all significantly related to one of the two perception dimensions and preference. Gender was usually thought to influence safety (Jansson et al., 2013), and women potentially seek more security, use less green space (Jim and Shan, 2013; Liu et al., 2022) and may feel a greater sense of wildness and less safety than men when faced with the same ground cover type. In addition, the elderly population was thought to care less about security which may originate from their childhood experiences – in the early years when urbanization was not yet developed, their childhood had more contact with nature (Jim and Shan, 2013). Thus, the elderly population may have felt less wild than participants of other age groups, especially teenagers under 18 who mainly rely on urban parks for exposure to nature. As opposed to previous reports that people who are better educated preferred natural landscape such as spontaneous vegetation (Zheng et al., 2011), the participants who had obtained a master’s degree perceived a lower ecological aesthetic value and showed lower preferences, as well as participants with junior high school education. Furthermore, people engaged in other occupations (mostly housewives looking after children) showed a higher preference. These results implied people with higher education levels may occupy higher demands for ground covers in ecology and aesthetic attributes, while “average,” or ordinary people find it easier to meet their personal requirements for ground covers. Thus, in order to approve the utilization of spontaneous groundcover in urban green spaces, it is necessary to reflect the ecological aesthetic value of the spontaneous groundcover for highly educated residents and professionals, and to provide enough exposure opportunities for ordinary, or “average,” people.

While monthly income and living environments did not exhibit significant influence on ground cover perceptions in our study, previous studies showed that higher economic status may contribute to the priority to obtain more social space due to their higher private car ownership rate (Zhang et al., 2019; Tao et al., 2020), which then results in a difference in green space use pattern and perception. In this study, since Xianyang is a city with a moderate economic level, the green spaces there were distributed fairly homogeneously. Thus, the inhabitants with different incomes do not obtain green spaces with extreme differences.

Living environment was demonstrated not to affect preference for vegetated landscape (Wang and Zhao, 2017). The increase of population mobility between urban and rural areas and the homogenization of urban and rural landscape can be seen to promote the familiarity and perception of people in different living environments concerning spontaneous groundcovers and lawns.

### 4.5. Limitations and further study recommendations

Even though different ground covers were selected in winter and spring to show the most obvious seasonal appearance of ground covers, it may not have been enough for participants to recognize them. Evidences have proved that preference is significantly related to seasonality (Southon et al., 2017; Xiang et al., 2021). To glean a more comprehensive understanding of perceptions and preferences, future research should contemplate evaluating ground covers across all seasons.

Considering that only photographs of quadrats sized 2 m × 2 m were used in the questionnaire survey, it may have been a challenge for the participants to experience the actual ground covers. Although the context was controlled in the experimental photos, more technology (like virtual reality) could be used to assist in building the same and living contexts, in order to better explore humans’ attitudes toward ground covers.

## 5. Conclusion

Due to the limitations of urban lawns, it is of great importance to explore the possibility of sustainable and enjoyable lawn alternatives. This study compared the perceptions and preferences concerning urban lawns and spontaneous ground covers and found there were no differences in preferences for the two types of ground cover. The *T. mongolicum* – *C. dactylon – C. canadensis* community was significantly preferred most among the 13 communities. Participants perceived spontaneous groundcovers as more natural, wild, varied, and species-richer compared to conventional lawns, while lawns were better kept than spontaneous ground covers. Industry and the degree of attention to herbaceous plants mostly affected the perception and preference among the social background factors measured. Gender, age, occupation, and education level also affected one of the perception dimensions and preference, whereas monthly income and living environment did not exert notable effects.

The results provide valuable insights for groundcover selection from the perspective of the acceptance of residents, showing that spontaneous groundcover has the potential to become a substitute for urban lawn usage in an undeveloped city but the type should be considered carefully. For landscape design in Xianyang, the community with dominant species *T. mongolicum*, *C. dactylon*, and *C. canadensis* is particularly recommended in ground cover application. Landscape architects should strategically guide plant succession to foster communities enriched with these dominant species and diverse flora, enhancing their ecological and aesthetic appeal to residents. At the same time, people preferred ground covers with greater aesthetic and ecology values but less characteristics of wildness, indicating the spontaneous groundcover can be used in spaces loose in maintenance requirements.

## Data availability statement

The original contributions presented in this study are included in this article/Supplementary material, further inquiries can be directed to the corresponding authors.

## Ethics statement

The studies involving humans were approved by the Ethics Committee of the College of Landscape Architecture and Arts, Northwest A&F University. The studies were conducted in accordance with the local legislation and institutional requirements. Written informed consent for participation in this study was provided by the participants’ legal guardians/next of kin.

## Author contributions

HL: Conceptualization, Data curation, Formal analysis, Methodology, Visualization, Writing — original draft. CL: Conceptualization, Data curation, Formal analysis, Methodology, Visualization, Writing — original draft. DX: Conceptualization, Data curation, Investigation, Writing — original draft. JL: Conceptualization, Data curation, Investigation, Writing — original draft. KJ: Conceptualization, Data curation, Investigation, Writing — original draft. YW: Conceptualization, Data curation, Investigation, Writing — original draft. DG: Conceptualization, Data curation, Investigation, Writing — original draft. YC: Conceptualization, Data curation, Investigation, Writing — original draft. YL: Conceptualization, Project administration, Supervision, Writing — original draft. LQ: Conceptualization, Funding acquisition, Methodology, Project administration, Resources, Writing — review and editing. TG: Conceptualization, Funding acquisition, Methodology, Project administration, Resources, Writing — review and editing.
