# Supplementary material for: Lawn or spontaneous groundcover? Residents’ perceptions of and preferences for alternative lawns in Xianyang, China
Source: Front Psychol. 2023 Oct 27;14:1259920. doi: 10.3389/fpsyg.2023.1259920 (PMC10646185; doi:10.3389/fpsyg.2023.1259920)
Supplement: Supplementary file 1 [file Data_Sheet_1.docx]

Supplementary Material

# Supplementary Tables

| Table 1. Social background characteristics of the participants | | | |
| --- | --- | --- | --- |
| **Social background Characteristics** | **Variable** | **Number of participants** | **Percentage of participants (%)** |
| **Gender** | Male | 1858 | 44.90 |
|  | Female | 2280 | 55.10 |
| **Age** | Below 18 | 303 | 7.32 |
|  | 18-22 | 748 | 18.08 |
|  | 23-30 | 996 | 24.07 |
|  | 31-40 | 956 | 23.10 |
|  | 41-50 | 509 | 12.30 |
|  | 51-60 | 377 | 9.11 |
|  | Over 60 | 249 | 6.02 |
| **Education level** | Primary school and lower | 371 | 8.97 |
|  | Junior high school | 456 | 11.02 |
|  | Senior high school | 640 | 15.47 |
|  | Undergraduate/ College | 2114 | 51.09 |
|  | Master | 458 | 11.07 |
|  | Ph.D. | 99 | 2.39 |
| **Monthly income** | ＜1000￥ | 1289 | 31.15 |
|  | 1000-3000￥ | 766 | 18.51 |
|  | 3000-5000￥ | 979 | 23.66 |
|  | 5000-10000￥ | 812 | 19.62 |
|  | 10000-20000￥ | 202 | 4.88 |
|  | 20000-30000￥ | 335 | 8.10 |
|  | 30000-50000￥ | 21 | 0.51 |
|  | >50000￥ | 34 | 0.82 |
| **Occupation** | Farmer | 272 | 6.57 |
|  | Off-farm worker | 111 | 2.68 |
|  | Business manager | 258 | 6.23 |
|  | Front-line employee or technical personnel of the enterprise | 494 | 11.94 |
|  | Institutional staff | 507 | 12.25 |
|  | Civil servant | 171 | 4.13 |
|  | Private owner | 230 | 5.56 |
|  | Freelancer | 384 | 9.28 |
|  | Student | 1237 | 29.89 |
|  | Unemployed | 168 | 4.06 |
|  | Others | 306 | 7.39 |
| **Industry** | Students or practitioners majoring in agriculture and forestry/ecology/environmental science/landscape architecture | 419 | 10.13 |
|  | Enthusiasts in agriculture and forestry/ecology/environmental science/landscape architecture | 465 | 11.24 |
|  | None of the above | 3254 | 78.64 |
| **Living environment** | Country | 1030 | 24.89 |
|  | City | 3108 | 75.11 |
| **Concerns of grass** | Often pay attention to grass in everyday life | 1146 | 27.69 |
|  | Occasionally pay attention to grass in everyday life | 2634 | 63.65 |
|  | Absolutely pay attention to grass in everyday life | 358 | 8.65 |

Table 2. Total variance explained by the components

| Component | Initial eigenvalue | | | Extraction sums of squared loadings | | | Rotation sums of squared loadings | | |
| --- | --- | --- | --- | --- | --- | --- | --- | --- | --- |
|  | Sum | Variation% | Accumulation% | Sum | Variation% | Accumulation% | Sum | Variation% | Accumulation% |
| 1 | 3.462 | 43.273 | 43.273 | 3.462 | 43.273 | 43.273 | 3.288 | 41.106 | 41.106 |
| 2 | 1.414 | 17.669 | 60.942 | 1.414 | 17.669 | 60.942 | 1.587 | 19.837 | 60.942 |
| 3 | 0.731 | 9.137 | 70.080 |  |  |  |  |  |  |
| 4 | 0.577 | 7.215 | 77.295 |  |  |  |  |  |  |
| 5 | 0.527 | 6.582 | 83.877 |  |  |  |  |  |  |
| 6 | 0.496 | 6.204 | 90.081 |  |  |  |  |  |  |
| 7 | 0.438 | 5.474 | 95.555 |  |  |  |  |  |  |
| 8 | 0.356 | 4.445 | 100.000 |  |  |  |  |  |  |

Table 3. Perception on *ecological aesthetics* across social background groups

| Factor | Variable | B | S.E. | 95% Wald confidence interval | | | Wald | df | Sig. |
| --- | --- | --- | --- | --- | --- | --- | --- | --- | --- |
|  |  |  |  | minimum | | maximum |  |  |  |
| Gender | Male | .005 | .0322 | -.058 | .068 | | 1.693 |  | 0.193 |
|  | Female | 0^a^ | . | . | . | | . | . | . |
| Age | Below 18 | .182 | .1088 | -.031 | .395 | | 2.797 | 1 | .094 |
|  | 18-22 | .006 | .0931 | -.176 | .189 | | .005 | 1 | .944 |
|  | 23-30 | -.064 | .0803 | -.222 | .093 | | .640 | 1 | .424 |
|  | 31-40 | -.081 | .0768 | -.232 | .069 | | 1.125 | 1 | .289 |
|  | 41-50 | -.050 | .0804 | -.208 | .108 | | .386 | 1 | .535 |
|  | 51-60 | .059 | .0814 | -.100 | .219 | | .531 | 1 | .466 |
|  | Over 60 | 0^a^ | . | . | . | | . | . | . |
| Education level | Primary school and lower | .168 | .1272 | -.082 | .417 | | 1.738 | 1 | .187 |
|  | Junior high school | .085 | .1209 | -.152 | .322 | | .495 | 1 | .482 |
|  | Senior high school | .176 | .1145 | -.048 | .401 | | 2.366 | 1 | .124 |
|  | Undergraduate/ College | .121 | .1056 | -.086 | .328 | | 1.317 | 1 | .251 |
|  | Master | -.040 | .1101 | -.255 | .176 | | .129 | 1 | .719 |
|  | Ph.D. | 0^a^ | . | . | . | | . | . | . |
| Occupation | Farmer | -.206 | .0880 | -.378 | -.033 | | 5.464 | 1 | .019 |
|  | Off-farm worker | -.286 | .1120 | -.506 | -.066 | | 6.512 | 1 | .011 |
|  | Business manager | -.064 | .0854 | -.231 | .104 | | .555 | 1 | .456 |
|  | Front-line employee or technical personnel of the enterprise | -.172 | .0717 | -.313 | -.032 | | 5.764 | 1 | .016 |
|  | Institutional staff | -.131 | .0743 | -.276 | .015 | | 3.089 | 1 | .079 |
|  | Civil servant | -.151 | .0961 | -.339 | .038 | | 2.454 | 1 | .117 |
|  | Private owner | -.180 | .0868 | -.350 | -.009 | | 4.278 | 1 | .039 |
|  | Freelancer | -.157 | .0749 | -.303 | -.010 | | 4.370 | 1 | .037 |
|  | Student | -.214 | .0846 | -.380 | -.048 | | 6.411 | 1 | .011 |
|  | Unemployed | -.326 | .0983 | -.519 | -.134 | | 11.022 | 1 | .001 |
|  | Others | 0^a^ | . | . | . | | . | . | . |
| Monthly income | ＜1000￥ | -.035 | .1742 | -.376 | .307 | | .040 | 1 | .842 |
|  | 1000-3000￥ | -.020 | .1724 | -.358 | .318 | | .014 | 1 | .907 |
|  | 3000-5000￥ | -.041 | .1709 | -.376 | .294 | | .058 | 1 | .809 |
|  | 5000-10000￥ | -.074 | .1709 | -.409 | .261 | | .185 | 1 | .667 |
|  | 10000-20000￥ | -.161 | .1806 | -.515 | .193 | | .795 | 1 | .373 |
|  | 20000-30000￥ | -.004 | .2346 | -.464 | .455 | | .000 | 1 | .985 |
|  | 30000-50000￥ | .356 | .2698 | -.173 | .885 | | 1.741 | 1 | .187 |
|  | >50000￥ | 0^a^ | . | . | . | | . | . | . |
| Industry | Students or practitioners majoring in agriculture and forestry/ecology/environmental science/landscape architecture | -.181 | .0541 | -.287 | -.075 | | 11.216 | 1 | .001 |
|  | Enthusiasts in agriculture and forestry/ecology/environmental science/landscape architecture | -.070 | .0504 | -.168 | .029 | | 1.910 | 1 | .167 |
|  | None of the above | 0^a^ | . | . | . | | . | . | . |
| Living environment | Country | -.019 | .0378 | -.093 | .055 | | .248 | 1 | .619 |
|  | City | 0^a^ | . | . | . | | . | . | . |
| Concerns of grass | Often pay attention to grass in everyday life | .646 | .0608 | .527 | .766 | | 112.930 | 1 | .000 |
|  | Occasionally pay attention to grass in everyday life | .387 | .0567 | .275 | .498 | | 46.477 | 1 | .000 |
|  | Absolutely not pay attention to grass in everyday life | 0^a^ | . | . | . | | . | . | . |

Table 4. Perception on *wildness* across social background groups

| Factor | Variable | B | S.E. | 95% Wald confidence interval | | Wald | df | Sig. |
| --- | --- | --- | --- | --- | --- | --- | --- | --- |
|  |  |  |  | minimum | maximum |  |  |  |
| Gender | Male | -.130 | .0322 | -.193 | -.067 | 16.203 | 1 | .000 |
|  | Female | 0^a^ | . | . | . | . | . | . |
| Age | Below 18 | .312 | .1088 | .098 | .525 | 8.208 | 1 | .004 |
|  | 18-22 | .233 | .0930 | .051 | .416 | 6.282 | 1 | .012 |
|  | 23-30 | .218 | .0802 | .061 | .375 | 7.378 | 1 | .007 |
|  | 31-40 | .306 | .0768 | .156 | .457 | 15.893 | 1 | .000 |
|  | 41-50 | .271 | .0804 | .113 | .428 | 11.324 | 1 | .001 |
|  | 51-60 | .224 | .0813 | .065 | .384 | 7.590 | 1 | .006 |
|  | Over 60 | 0^a^ | . | . | . | . | . | . |
| Education level | Primary school and lower | -.011 | .1271 | -.260 | .238 | .007 | 1 | .933 |
|  | Junior high school | -.115 | .1209 | -.352 | .122 | .900 | 1 | .343 |
|  | Senior high school | -.012 | .1145 | -.237 | .212 | .011 | 1 | .915 |
|  | Undergraduate/ College | .014 | .1056 | -.193 | .221 | .018 | 1 | .894 |
|  | Master | -.088 | .1100 | -.303 | .128 | .635 | 1 | .425 |
|  | Ph.D. | 0^a^ | . | . | . | . | . | . |
| Occupation | Farmer | .051 | .0879 | -.121 | .224 | .339 | 1 | .560 |
|  | Off-farm worker | -.166 | .1120 | -.385 | .054 | 2.193 | 1 | .139 |
|  | Business manager | -.237 | .0854 | -.404 | -.070 | 7.701 | 1 | .006 |
|  | Front-line employee or technical personnel of the enterprise | -.097 | .0717 | -.238 | .043 | 1.846 | 1 | .174 |
|  | Institutional staff | -.246 | .0743 | -.392 | -.100 | 10.962 | 1 | .001 |
|  | Civil servant | -.219 | .0961 | -.407 | -.031 | 5.193 | 1 | .023 |
|  | Private owner | -.104 | .0868 | -.274 | .066 | 1.436 | 1 | .231 |
|  | Freelancer | -.050 | .0749 | -.197 | .097 | .446 | 1 | .504 |
|  | Student | -.030 | .0846 | -.196 | .136 | .126 | 1 | .723 |
|  | Unemployed | -.029 | .0983 | -.222 | .164 | .087 | 1 | .769 |
|  | Others | 0^a^ | . | . | . | . | . | . |
| Monthly income | ＜1000￥ | -.305 | .1741 | -.646 | .036 | 3.068 | 1 | .080 |
|  | 1000-3000￥ | -.312 | .1723 | -.650 | .026 | 3.281 | 1 | .070 |
|  | 3000-5000￥ | -.177 | .1708 | -.512 | .157 | 1.080 | 1 | .299 |
|  | 5000-10000￥ | -.240 | .1709 | -.575 | .095 | 1.977 | 1 | .160 |
|  | 10000-20000￥ | -.311 | .1806 | -.665 | .043 | 2.962 | 1 | .085 |
|  | 20000-30000￥ | -.354 | .2345 | -.814 | .106 | 2.280 | 1 | .131 |
|  | 30000-50000￥ | -.309 | .2697 | -.837 | .220 | 1.308 | 1 | .253 |
|  | >50000￥ | 0^a^ | . | . | . | . | . | . |
| Industry | Students or practitioners majoring in agriculture and forestry/ecology/environmental science/landscape architecture | .005 | .0541 | -.101 | .111 | .007 | 1 | .932 |
|  | Enthusiasts in agriculture and forestry/ecology/environmental science/landscape architecture | -.125 | .0504 | -.224 | -.026 | 6.135 | 1 | .013 |
|  | None of the above | 0^a^ | . | . | . | . | . | . |
| Living environment | Country | -.035 | .0378 | -.109 | .039 | .867 | 1 | .352 |
|  | City | 0^a^ | . | . | . | . | . | . |
| Concerns of grass | Often pay attention to grass in everyday life | -.132 | .0608 | -.251 | -.013 | 4.698 | 1 | .030 |
|  | Occasionally pay attention to grass in everyday life | -.032 | .0567 | -.143 | .079 | .313 | 1 | .576 |
|  | Absolutely not pay attention to grass in everyday life | 0^a^ | . | . | . | . | . | . |

Table 5. Preferences for ground covers across social background groups.

| Factor | Variable | B | S.E. | 95% Wald confidence interval | | Wald | df | Sig. |
| --- | --- | --- | --- | --- | --- | --- | --- | --- |
|  |  |  |  | minimum | maximum |  |  |  |
| Gender | Male | .083 | .0323 | .019 | .146 | 6.523 | 1 | .011 |
|  | Female | 0^a^ | . | . | . | . | . | . |
| Age | Below 18 | .254 | .1092 | .040 | .468 | 5.425 | 1 | .020 |
|  | 18-22 | .088 | .0934 | -.095 | .271 | .893 | 1 | .345 |
|  | 23-30 | -.008 | .0806 | -.165 | .150 | .009 | 1 | .926 |
|  | 31-40 | -.075 | .0771 | -.226 | .076 | .953 | 1 | .329 |
|  | 41-50 | -.059 | .0807 | -.217 | .100 | .527 | 1 | .468 |
|  | 51-60 | .035 | .0817 | -.125 | .195 | .187 | 1 | .665 |
|  | Over 60 | 0^a^ | . | . | . | . | . | . |
| Education level | Primary school and lower | .077 | .1276 | -.173 | .327 | .364 | 1 | .546 |
|  | Junior high school | -.053 | .1214 | -.290 | .185 | .188 | 1 | .665 |
|  | Senior high school | .161 | .1149 | -.064 | .386 | 1.961 | 1 | .161 |
|  | Undergraduate/ College | .029 | .1060 | -.179 | .237 | .074 | 1 | .785 |
|  | Master | -.070 | .1105 | -.286 | .147 | .400 | 1 | .527 |
|  | Ph.D. | 0^a^ | . | . | . | . | . | . |
| Occupation | Farmer | -.201 | .0883 | -.374 | -.028 | 5.184 | 1 | .023 |
|  | Off-farm worker | -.252 | .1125 | -.472 | -.032 | 5.020 | 1 | .025 |
|  | Business manager | -.049 | .0858 | -.217 | .119 | .322 | 1 | .571 |
|  | Front-line employee or technical personnel of the enterprise | -.143 | .0720 | -.284 | -.002 | 3.929 | 1 | .047 |
|  | Institutional staff | -.081 | .0746 | -.227 | .065 | 1.188 | 1 | .276 |
|  | Civil servant | -.141 | .0965 | -.330 | .048 | 2.134 | 1 | .144 |
|  | Private owner | -.176 | .0871 | -.347 | -.006 | 4.105 | 1 | .043 |
|  | Freelancer | -.177 | .0751 | -.324 | -.029 | 5.520 | 1 | .019 |
|  | Student | -.268 | .0849 | -.435 | -.102 | 9.963 | 1 | .002 |
|  | Unemployed | -.300 | .0987 | -.493 | -.106 | 9.218 | 1 | .002 |
|  | Others | 0^a^ | . | . | . | . | . | . |
| Monthly income | ＜1000￥ | .095 | .1748 | -.247 | .438 | .296 | 1 | .586 |
|  | 1000-3000￥ | .110 | .1730 | -.230 | .449 | .401 | 1 | .527 |
|  | 3000-5000￥ | .046 | .1715 | -.290 | .382 | .071 | 1 | .790 |
|  | 5000-10000￥ | -.013 | .1715 | -.349 | .324 | .005 | 1 | .941 |
|  | 10000-20000￥ | -.057 | .1813 | -.412 | .298 | .099 | 1 | .753 |
|  | 20000-30000￥ | .107 | .2354 | -.355 | .568 | .206 | 1 | .650 |
|  | 30000-50000￥ | .460 | .2708 | -.071 | .991 | 2.884 | 1 | .089 |
|  | >50000￥ | 0^a^ | . | . | . | . | . | . |
| Industry | Students or practitioners majoring in agriculture and forestry/ecology/environmental science/landscape architecture | -.177 | .0543 | -.283 | -.070 | 10.594 | 1 | .001 |
|  | Enthusiasts in agriculture and forestry/ecology/environmental science/landscape architecture | -.165 | .0506 | -.264 | -.066 | 10.644 | 1 | .001 |
|  | None of the above | 0^a^ | . | . | . | . | . | . |
| Living environment | Country | .017 | .0380 | -.058 | .091 | .189 | 1 | .664 |
|  | City | 0^a^ | . | . | . | . | . | . |
| Concerns of grass | Often pay attention to grass in everyday life | .685 | .0610 | .565 | .805 | 125.948 | 1 | .000 |
|  | Occasionally pay attention to grass in everyday life | .436 | .0569 | .325 | .548 | 58.824 | 1 | .000 |
|  | Absolutely not pay attention to grass in everyday life | 0^a^ | . | . | . | . | . | . |

# Questionnaire

1. What is your impression of these pictures?

1-5 means the transition from the left adjective to the right adjective, for example: 1 means the most "artificial," 5 means the most "natural."
*Note: "Domesticated" refers to the selection and cultivation of wild animals or plants, which gradually change their original habits and become livestock, poultry or cultivated plants that can meet people's needs. For example, wolves have been domesticated for a long time to produce dogs; corn, wheat and tomatoes are domesticated from wild species.

|  | 1 | 2 | 3 | 4 | 5 |  |
| --- | --- | --- | --- | --- | --- | --- |
| Artificial | ○ | ○ | ○ | ○ | ○ | Natural |
| Domesticated^*^ | ○ | ○ | ○ | ○ | ○ | Wild |
| Uninteresting | ○ | ○ | ○ | ○ | ○ | Interesting |
| Ugly | ○ | ○ | ○ | ○ | ○ | Beautiful |
| Neglected | ○ | ○ | ○ | ○ | ○ | Well kept |
| Monotonous | ○ | ○ | ○ | ○ | ○ | Varied |
| Non-ecological | ○ | ○ | ○ | ○ | ○ | Ecological |
| With few species | ○ | ○ | ○ | ○ | ○ | With rich species |

2. How do you like these pictures?

| very dislike | ○1 | ○2 | ○3 | ○4 | ○5 | very like |
| --- | --- | --- | --- | --- | --- | --- |

3. What is your reason for making this choice? _________________________________

4. Your gender:

| ○Male | ○Female |
| --- | --- |

5. Your age: _________________________________

6. What is your educational background?

| ○Primary school and lower | ○Junior high school | ○Senior high school |
| --- | --- | --- |
| ○Undergraduate/College | ○Master | ○Ph. D. |

7. What is your occupation?

| ○Farmer | ○Off-farm worker | ○Business manager |
| --- | --- | --- |
| ○Front-line employee or technical personnel of the enterprise (workers, service personnel, technicians, lawyers, etc.) | | |
| ○Institutional staff (including doctors, teachers, etc.) | | |
| ○Civil servant | ○Private owner | ○Freelancer |
| ○Student | ○Unemployed | ○Others _________________ |

8. What is your monthly income?

Fill in according to the actual income. If the student has no income, select the first item.

| ○＜1000￥ | ○1000-3000￥ | ○3000-5000￥ | ○5000-10000￥ |
| --- | --- | --- | --- |
| ○10000-20000￥ | ○20000-30000￥ | ○30000-50000￥ | ○>50000￥ |

9. What is your major?

| ○Students or Practitioners majoring in agriculture and forestry/ecology/environmental science/landscape architecture |
| --- |
| ○Enthusiasts in agriculture and forestry/ecology/environmental science/landscape architecture |
| ○None of the above |

10. What is your main living environment so far?

| ○Country | ○City |
| --- | --- |

11. Have you ever paid attention to the grass around you in your everyday life?

| ○Often | ○Occasionally | ○Absolutely not |
| --- | --- | --- |
